# Platelet extracts induce growth, migration and invasion in human hepatocellular carcinoma in vitro

**DOI:** 10.1186/1471-2407-14-43

**Published:** 2014-01-27

**Authors:** Brian I Carr, Aldo Cavallini, Rosalba D’Alessandro, Maria Grazia Refolo, Catia Lippolis, Antonio Mazzocca, Caterina Messa

**Affiliations:** 1 Laboratory of biochemistry and tumor biology, National Institute for Digestive Diseases, IRCCS ‘Saverio de Bellis’, via Turi 27, 70013 Castellana Grotte, BA, Italy; 2 Department of Emergency and Organ Transplantation, Section of Internal Medicine, University of Bari, piazza G. Cesare, 70100 Bari, Italy

**Keywords:** Platelets, HCC, Growth, Migration, Invasion, AFP

## Abstract

**Background:**

Thrombocytopenia has been reported to be associated with small size HCCs, and thrombocytosis to be associated with large size HCCs. The aim was to examine the effects of platelets in relation to HCC cell growth.

**Methods:**

The effects of time-expired pooled normal human platelets were examined on human HCC cell line growth and invasion.

**Results:**

Blood platelet numbers increased with increasing HCC tumor size and portal vein invasion. Platelet extracts enhanced cell growth in 4 human HCC cell lines, as well as cell migration, medium AFP levels and decreased apoptosis. Cell invasion was significantly enhanced, using a Matrigel-coated trans-well membrane and3D (Real-Time Imaging) invasion assay. Western blots showed that platelets caused enhanced phospho-ERK and phospho–JNK signaling and anti-apoptotic effect with increase of Bcl-xL (anti-apoptotic marker) and decrease of Bid (pro-apoptotic marker) levels. Their growth effects were blocked by a JNK inhibitor.

**Conclusions:**

Platelets stimulated growth and invasion of several HCC cell lines in vitro, suggesting that platelets or platelet growth factors could be a potential pharmacological target.

## Background

Platelets have a key function in blood clotting. However, it is increasingly recognized that they have other actions, including in cancer biology. Thrombocytosis has been reported to occur in association with solid tumors and over 40% of patients with thrombocytosis without iron deficiency anemia have occult metastasis, typically of the gastrointestinal system, breast, lung and ovary reviews: [1-4]. Cancer can result in altered coagulation and platelet activity and conversely, platelets have the ability to influence cancer growth and metastasis [2,5]. This can occur by direct platelet effects or through mesenchymal interactions [6,7].

Platelets have also been reported to enhance liver regenerative growth in animals and hepatocyte proliferation in vitro [8,9]. Conversely, thrombocytopenia can blunt regeneration [10]. Human hepatocellular carcinoma (HCC) typically arises on the basis of cirrhosis, most commonly caused by hepatitis B or C or alcoholism, exposure to food contamination by mycotoxins or to obesity. The fibrosis that is a key aspect of cirrhosis, eventually causes portal hypertension and associated splenomegaly, that is thought to cause subsequent thrombocytopenia. Thrombocytopenia of cirrhosis has recently been shown to be associated predominantly with small size HCCs [11], whereas very large size HCCs often have normal platelet counts [12] and thrombocytosis in HCC patients occurs most often with large size tumors [13]. We have therefore examined the effects of platelet extracts on the growth in vitro of human HCC cell lines and report that they enhance cell proliferation, migration and invasion.

## Methods

### Cells and materials

PLC/PRF/5, Hep3B and HepG2 cells were obtained from the ATCC and were cultured as we previously described [14].

### Platelet lysates

Apheresis platelets were collected from six healthy blood donors after obtaining their consent and the approval of the Ethics Committee of Institute “Saverio de Bellis” and University of Bari, Italy. The human platelet-rich plasma (PRP) was obtained using an automated haemapheresis procedure in a local blood transfusion center.

The platelets were subjected to several freeze-thaw cycles to disrupt their membranes and release the growth factors stored in the granules (human Platelet Lysate, hPL).

### Growth and migration assays

Proliferation and migration assays were performed as recently described [14]. The JNK inhibitor (SP600125; Selleck Chemicals, Houston, TX, USA) 20 μM was used to antagonize cell growth in presence of hPL or FBS.

### AFP measurement

Medium AFP levels were measured using an automated system (UniCel Integrated Workstations DxC 660i, Beckman Coulter, Fullerton, CA, USA) by a chemioluminescent immunometric method. Sample measurements over the calibration range were automatically re-analyzed according to manufacture’s instructions.

### Apoptosis assays

#### Annexin V

The Muse Annexin V/Dead Cell Assay Kit (Millipore, Darmstadt, Germany) for quantitative analysis of live, early/late apoptotic and dead cells was used with a Muse Cell Analyzer (Millipore). Briefly, the assay utilizes Annexin V to detect PS on the external membrane of apoptotic cells. A dead cell marker (7-AAD) is also used. PLC/PRF/5 cell line, including positive and negative controls, were cultured in 1% FBS medium supplemented with a volume of hPL corresponding to 3.75×10^7^platelets/ml or with an equivalent percentage of serum (control cells) for 48 h. The cells were then processed as described in the user’s guide.

#### Caspase-3/7 quantitative measurements

The Muse Caspase-3/7 kit (Millipore) permits simultaneous evaluation of apoptotic status based on Caspase-3 and -7 activation and cellular plasma membrane permeabilization (cell death).

The assay provides relative percentage of cells that are live, early/late apoptotic or dead. Cells were cultured as described above and processed according to the user’s guide.

### Matrigel invasion assay

Huh7-GFP cells were generated by infection with retroviral particles containing pLXSN-GFP vector (Clontech Laboratories, Mountain View, CA, USA) and isolated by neomycin selection without clonal propagation.

Invasion was performed as previously described [15]. Briefly, 8 μm trans-well membranes (Corning Life Sciences, Manassas, VA, USA) were pre-coated with 20 μg/ml BD Matrigel™ Basement Membrane Matrix (BD Biosciences, Buccinasco (MI), Italy).

Huh7-GFP cells were trypsinized and loaded (1 × 10^5^ cells) into the upper chamber of the trans-well plates and allowed to invade for 24 h. After fixation with 4% paraformaldehyde, invaded cells were quantified by counting the GFP-positive cells.

### Real-time imaging of the 3D Matrigel invasion

For 3D experiments, Huh7-GFP were trypsinized, counted and seeded on the top of the lower polymerized Matrigel layer and allowed to adhere and spread. After 3 h, the same cold Matrigel solution was added to cover the cells and to form the upper layer of the 3D Matrigel. Following polymerization, an appropriate dilution of hPL in DMEM medium or DMEM containing 1% BSA was added to the wells. The motility of invasive cells within the 3D Matrigel was monitored by real-time imaging with a modified epi-illumination Zeiss microscope (Zeiss, Oberkochen, Germany) equipped with a Hamamatsu CCD camera (ORCA-AG; Hamamatsu Photonics, Hamamatsu city, Japan). Digital images were acquired using AxioVision imaging software (Zeiss, Jena, Germany) and further processed using Photoshop software (Adobe, San Jose, CA, USA).

### Western blots

We analyzed the MAPK signaling and anti-apoptosis markers in PLC/PRF/5 cells treated with hPL by Western blot, exactly as previously described [14]. In brief, cells were washed twice with cold PBS and then lysed in RIPA buffer (Sigma-Aldrich, Milan; Italy). After quantization of protein concentration, equal amount of protein (50 μg) were resolved on SDS–PAGE and transferred to polyvinyldifluoride (PVDF) filters. The blots were blocked with 5% (w/v) nonfat dry milk for 2 h at room temperature and then probed with primary antibody overnight at 4°C.

The primary antibodies were directed against the following proteins: ERK and phospho-ERK (p-ERK), JNK and phospho-JNK (p-JNK), STAT3 and phospho-STAT3 (Tyr^705^, Ser^727^) (pSTAT3), phospho-p38 MAPK (p-p38) and p38 MAPK, AKT and phospho-AKT (p-AKT), Bid, Bcl-xL and β-actin (Cell Signaling, Beverly, MA, USA). Immunoreactive bands were visualized and analyzed usingenhanced chemiluminescence detection reagents (Cell Signaling, Beverly, MA, USA) and a chemiluminescence detection system (ChemiDoc XRS apparatus, Bio-Rad. Milan, Italy). Results were representative of 3 independent experiments.

### In vitro experimental data

The differences between two unmatched groups were evaluated by Mann–Whitney nonparametric test.

For multiple comparisons was used one-way Anova test followed by Dunnett's post test.

The computer software used was GraphPad Prism version 5.0.

P-values of <0.05 were considered statistically significant.

All experiments were done in triplicate and data are presented as mean ± standard deviation (mean ± SD).

## Results

### Platelets as a source of HCC growth stimulants

**Figure 1:**
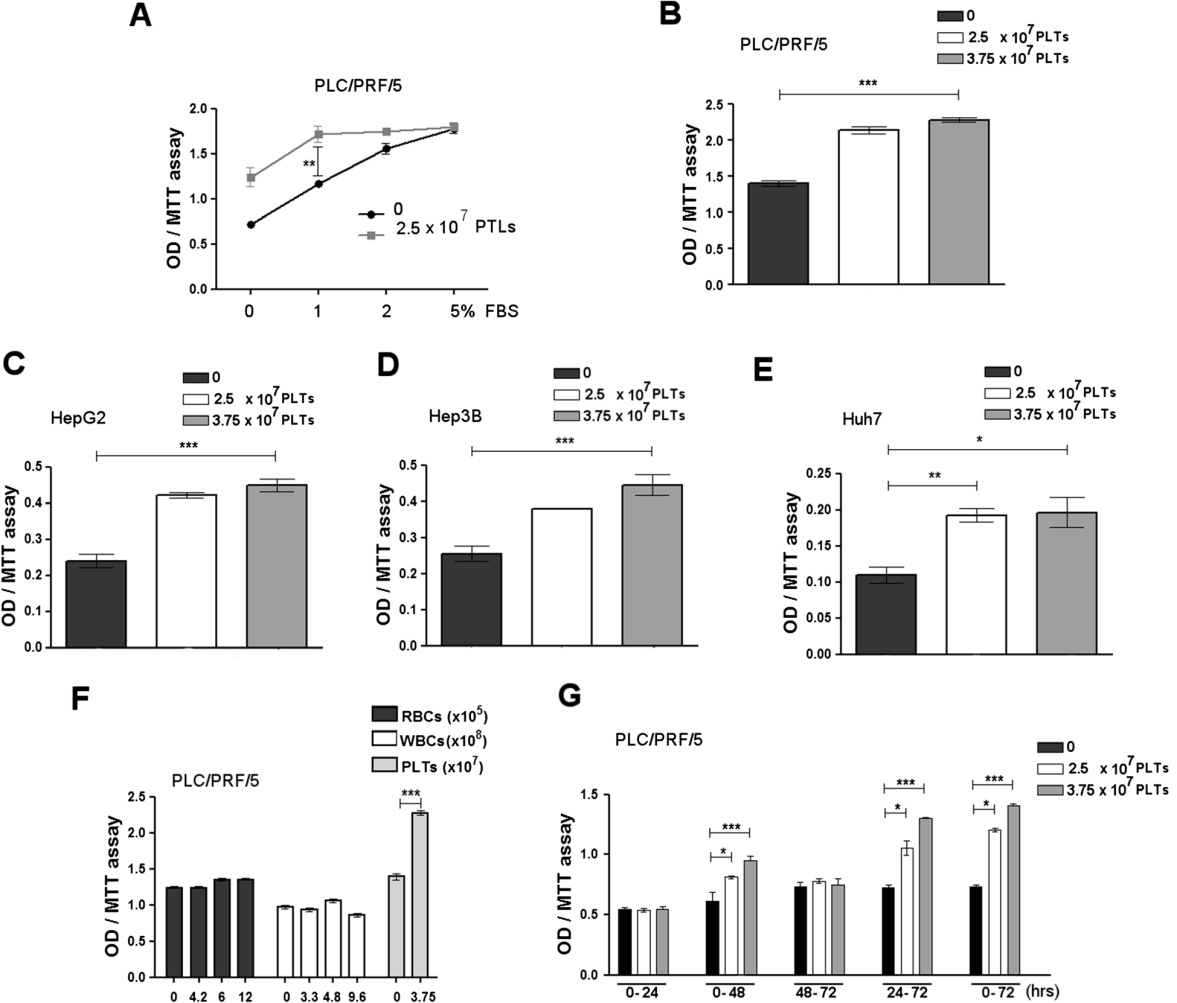
**Effects of platelet extracts on HCC cell line growth. (A)** Comparison of serum concentrations on platelet actions. PLC/RFP/5 cells were cultured in different FBS percentage (0-5%) for 48 h in presence of PLTs or equivalent FBS concentration (control). Performing MTT assay, a significant difference in growth is detected only at 1% FBS. **(B-E)** Effects of platelets on the growth of different human HCC cell lines. PLC/PRF/5 **(B)**, HepG2 **(C)**, Hep3B **(D)** and Huh7 **(E)** cell lines were cultured in 1% FBS medium in presence of different platelets concentrations or FBS and MTT assay was assessed after 48 h. **(F)** Comparison of different concentrations of red cells (RBC), white cells (WBC) and platelets on PLC/PRF/5 cell growth evaluated after 48 h by MTT assay. **(G)** Comparison of time windows (0-24 h, 0-48 h, 48-72 h, 24-72 h and 0-72 h) for effects of two different platelet concentrations on growth evaluated after 72 h by MTT assay. The results are expressed as mean ± SD. *P < 0.05; **P < 0.001; ***P < 0.0001.

hPL were examined for the ability to stimulate human HCC cell line growth. PLC/RFP/5 cells were cultured in different FBS concentrations (0-5%) for 48h in presence of hPL or equivalent FBS concentration (control) in order to define the minimum FBS concentration to maintain the health of cells without growth. Comparing the growth in presence of hPL or FBS by MTT assay, a significant difference is detected only at 1% FBS (Figure 1A). This low serum concentration was used in all further platelet lysate experiments, as higher serum concentrations caused maximum cell growth stimulation.

PLC/PRF/5, HepG2, Hep3B or Huh7-GFP cell lines were cultured in 1% FBS in presence of different hPL concentrations or FBS (control). Cell growth was enhanced by culture with hPL (Figure 1B-E). This growth stimulation was reversible, since sub-culture of the same treated cells without further addition of platelet extracts, resulted in a return to normal pre-treatment growth (data not shown).

### Characterization of hPL effects on cell growth

The effects of platelets on cell growth were examined further. Neither red cell (RBCs) nor white cell extracts (WBCs) had similar HCC growth stimulatory effects as platelets (Figure 1F).

**Figure 2:**
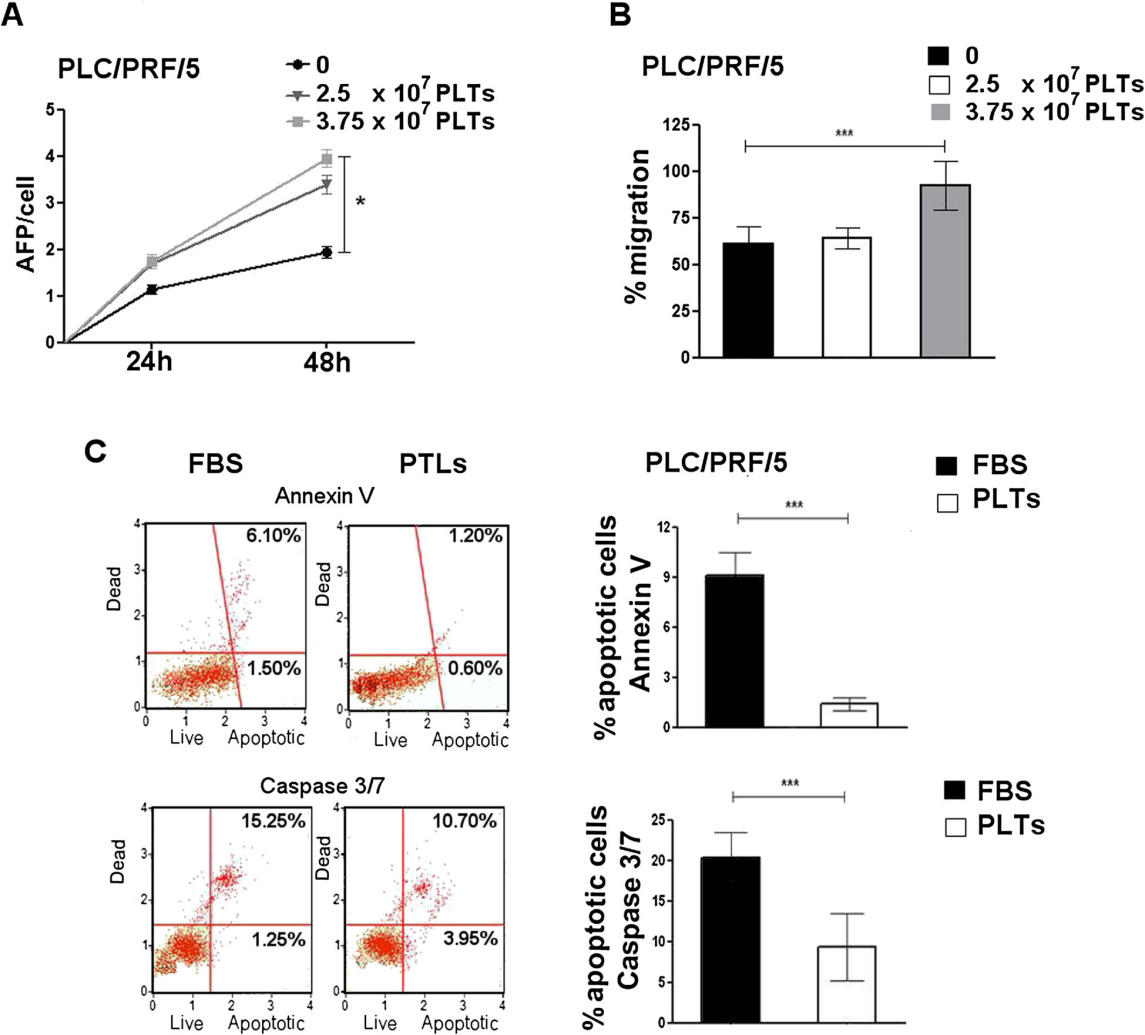
**Effects of platelets on PLC/PRF/5 cells. (A)** AFP levels in the cell culture medium of PLC/PRF/5 cell lines after treatment with different platelets concentrations or FBS. **(B)** Migration assay. PLC/PRF/5 cells were treated with different platelet concentrations or FBS and microscopically analyzed at the time of the scratch and after 72 h. Values were expressed as percentage of migration, 100% representing the completely closed wound. **(C)** Apoptosis assays. On the left are shown examples of results obtained using the Muse Annexin V kit (upper panel) or Caspase-3/7 kit (lower panel) to evaluate the percentage of apoptotic PLC/PRF/5 cells cultured whit PLTs or FBS. On the right the mean of three independent experiments is plotted in the relative graph. The results are expressed as mean ± SD. *P < 0.05; ***P < 0.0001.

To determine the time of exposure needed for platelets to enhance cell growth, cells were exposed to hPL for various time windows. A minimum of 48 h was found to be necessary (Figure 1G). Alpha-fetoprotein (AFP) is a secreted HCC protein that is commonly used as a measure of HCC growth and aggressiveness. We found that AFP levels in the cell culture medium were increased after exposure to hPL (Figure 2A). Since cell growth is a balance between growth stimulation and apoptosis, we then examined the percent of apoptotic cells in cultures with and without hPL and found that platelets decreased the baseline percent of apoptotic cells (Figure 2C). Cell migration was also investigated and also found to be enhanced by exposureto platelet extracts (Figure 2B).

### Platelet-enhancement of cell invasion

**Figure 3:**
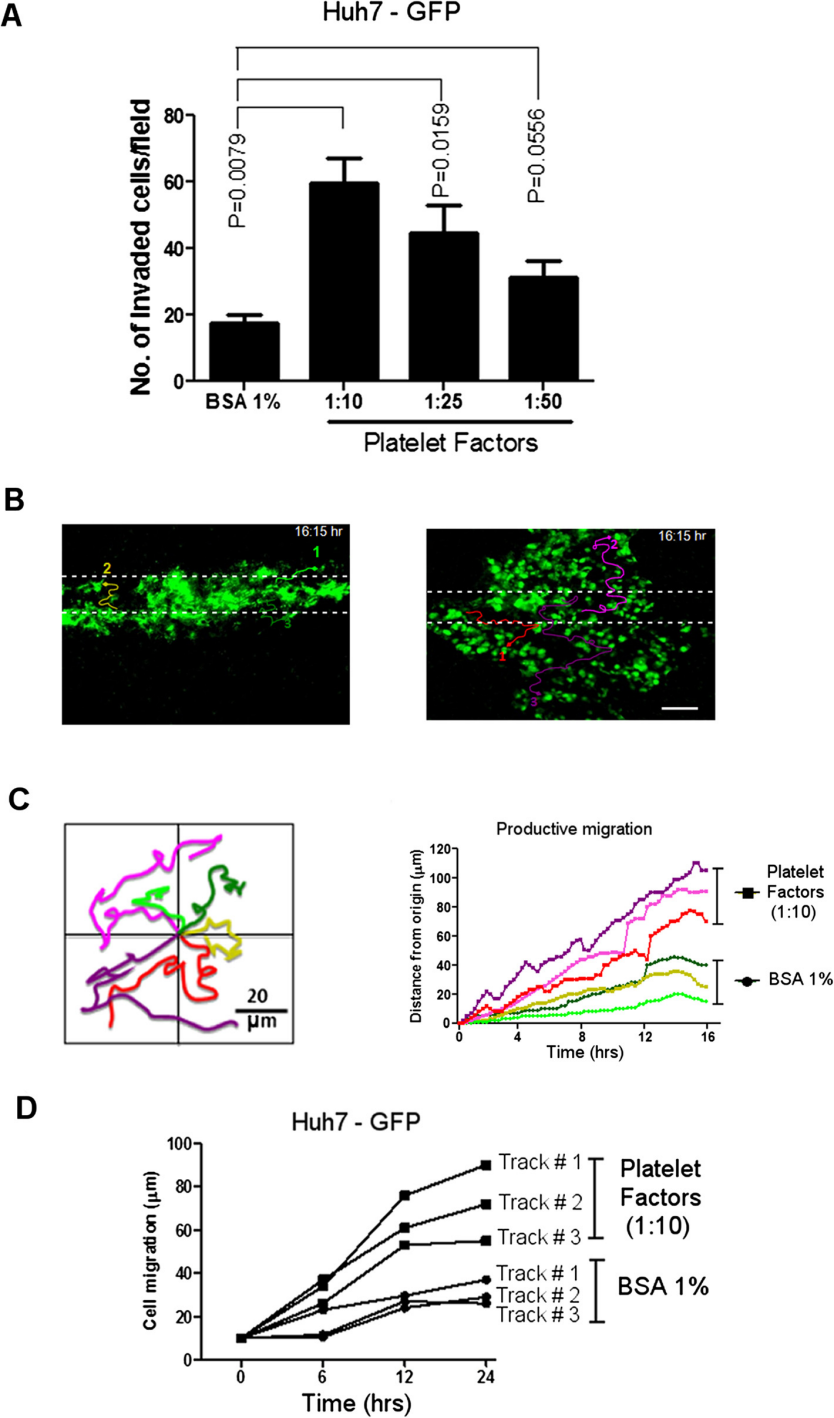
**Effects of platelets on hepatocellular carcinoma cell motility and invasion. (A)** Huh7-GFP cells were assayed by Matrigel chemo-invasion with BSA 1% (control) or different dilution of platelet factors added to a lower compartment of the chamber at the indicated concentrations. **(B)** Tracking the migration of Huh7-GFP cells by live optical imaging. GFP-expressing Huh7 were imaged for 16 h and the behavior of motile cells was recorded. **(C)** The tracks of 6 representative cells (3 controls and 3 stimulated with platelet factors) are plotted inWind-rose plots with the initial position of each track (left panel).Scale bar, 100 μm. The temporal increase in the productive motility of these representative tracks from controls and stimulated Huh7-GFP cells are also plotted in the graph (right panel). **(D)** 3D Invasion assay of Huh7-GFP cells in the presence of platelet soluble factors. The rate of cell migration was determined at different time intervals (6, 12, 24 h). Three representative tracks for each condition (cells stimulated with platelet factors or with BSA 1% as control) are plotted in the graph. All experiments were carried out in triplicate. The results are expressed as mean ± SEM. *P* values were determined by Mann–Whitney one-tailed test.

To further characterize a role for platelet factors in altering HCC tumor motility, we carried out 2D and 3D invasion assays using fluorescence labeled cells (Huh7-GFP). Using preliminary 2D transwell invasion experiments, we found that platelet factors increase tumor invasion of Huh7-GFP compared to control (BSA 1%) and that dilution is an important factor in determining the increased (Figure 3A). In fact, 1:10 dilution was the optimal concentration to determine the highest effect on tumor cell motility. This dose was used for the subsequent 3D experiments. The advantage of the 3D real-time imaging analysis is that individual cells could be tracked in real-time as shown in Figure 3B. By using this technique, we found an increase in cell motility in platelet factor-stimulated Huh7-GFP cells compared with controls and this was corroborated by the increase of productive migration (distance from the point of origin) in platelet factor-treated Huh7-GFP compared to controls (Figure 3C). The increased motility of individual tumor cells was also measured as distance traveled by the cells (expressed in μm) by the tracking of selected cells at different time intervals (Figure 3D).

### Mechanisms associated with growth stimulation by platelets

**Figure 4:**
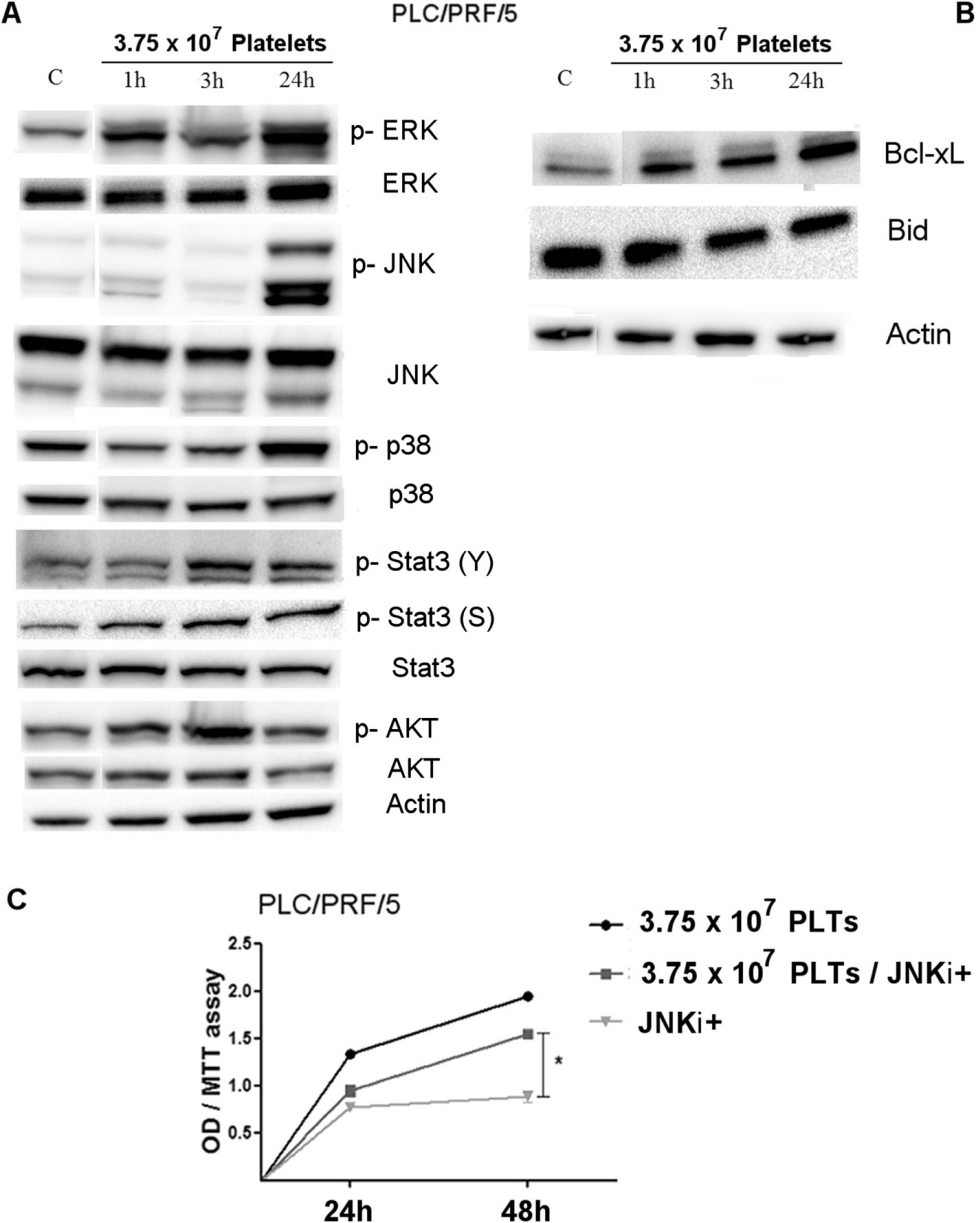
**Cell signaling changes induced by platelets. (A)** Analysis of changes in p-ERK, p-JNK, p-STAT3, p-p38 and p-AKT. PLC/PRF/5 cells were treated with 3.75 × 10^7^ PLTs or FBS for different times. The changes in protein expression were evaluated using Western blot (WB) analysis. **(B)** The Western blot (WB) analysis confirmed the anti-apoptotic effect of platelets as showed in Figure 2C. **(C)** To test the significance of p-JNK increase, observed in WB analysis **(A)**, on platelet-mediated growth induction, SP600125 (JNK inhibitor) was used, resulting in inhibition of PLT action. *P < 0.05.

Western blotting was performed on extracts of cells treated without (controls) or with hPL for common MAPK pathways associated with growth stimulation, and for anti-apoptosis mediators (Figure 4A-B). We found an increase in p-ERK levels, and especially in p-JNK levels, but not in p-p38 levels, together with an increase in p-STAT3 levels.

The anti-apoptotic effect was associated with two different mechanisms: an increase of Bcl-xL (anti-apoptotic marker) levels and a decrease of Bid (pro-apoptotic marker) levels. To test the significance of the strong p-JNK induction, cell growth enhancement by hPL was repeated in the presence or absence of a JNK inhibitor, which abrogated the stimulatory effects of hPL (Figure 4C).

## Discussion

There have been several studies of the interactions between blood platelets and tumor biology for several human cancers, including ovarian, breast and colon cancer [4,6,16,17]. Several mechanisms have been proposed to be involved, including altered cell adhesion, enhanced coagulation and platelet-derived inflammatory cytokines, angiogenesis factors and and/or tumor growth factors. Thus, platelet changes can occur in conjunction with coagulation changes in response to the growth of tumors, and conversely, platelets may be involved in tumor growth and metastasis [18,19]. To our knowledge, the current report is a first of a direct effect of platelets on HCC cell growth and invasion. We found that extracts of pooled normal human platelets stimulated growth of several human HCC cell lines in vitro, as well as cell migration and invasion. The effects were time-dependent and reversible, as subsequent sub-culture of platelet-treated cells without platelets, led to loss of the growth stimulant effects. Culture medium AFP levels were also increased with growth stimulation, and low baseline cell apoptosis levels were further reduced by exposure to platelet lysates. The clinical findings of enhanced incidence of portal vein thrombosis in the presence of larger tumors with higher platelet counts, led us to also study cell motility. Cell migration was increased using two different cellular models, an cell invasion was evaluated using Matrigel-treated membranes. The Western blot analysis showed an increase in p-ERK, p-STAT3 and especially p-JNK levels. A JNK inhibitor abrogated the growth stimulatory actions of the platelet extracts, showing the importance of this pathway in the platelet growth enhancing effects. Platelets and platelet-derived growth factors have been previously described to have effects on growth of hepatocytes [8-10,20-22].

Platelets and their products also influence HCC growth and biology [23-29], but a direct effect has not been previously reported and platelet-inhibition has recently been shown to antagonize hepatocarcinogenesis [30], likely in this model through modulation of necro-inflammation. Furthermore, experimental chemically induced hepatocarcinogenesis has been reported in association with carcinogen-induced platelet proteome changes [31]. Platelet factors that might be involved in HCC growth include inflammatory cytokines, Vascular endothelial growth factor (VEGF), Fibroblast growth factor (FGF), serotonin and Platelet-derived growth factors (PDGF).

Platelets and their extracts have been recently used therapeutically for their growth enhancing and wound healing effects [32-34], and they contain multiple growth factors including Insulin-like growth factor-1 (IGF-1), Epidermal growth factor (EGF), Transforming growth factor beta (TGFβ), PDGFs, FGFs, VEGFs, serotonin and interleukins. Identification of the platelet factors, which were involved in the growth stimulation reported here, is beyond the scope of this study. These same factors that have therapeutic healing potential for normal cells and tissues, can also worsen tumor growth. This has led to the evaluation of aspirin, a platelet modulator, both for its effects in inhibiting experimental HCC [30,34-37], but also in clinical trials for cancer risk reduction and metastasis reduction [37]. Platelet modulation has also been reported as affecting metastasis [20,38,39], and platelets have been shown to enhance cell migration and invasion [40,41]. Thus, the modulation of platelet function may have clinical application in both HCC prevention and in improving HCC biology, in patients without thrombocytopenia or other bleeding disorder.

## Conclusions

Extracts from normal human platelets, but not from red or white blood cells, could stimulate growth in vitro in several human HCC cell lines. The extracts also stimulated HCC cell migration and invasion. They inhibited apoptosis, by both decreasing apoptotic effectors and inducing anti-apoptotic mediators. p-JNK levels were elevated by hPL actions and JNK was a likely mediator of the hPL growth induction, since the growth increase was antagonized by addition of a JNK inhibitor to the hPL. Platelets therefore represent an additional potential micro-environmental factor in HCC cell growth.

## Abbreviations

HCC: Hepatocellular carcinoma; hPL: Human Platelets lysates; PVT: Portal vein thrombosis; ERK: Extracellular signal-regulated kinase; JNK: c-Jun NH2-terminal kinase; STAT: Signal transducer and activator of transcription-3; WB: Western blot; MTT: 3-(4,5-Dimethylthiazol-2-yl)-2,5-diphenyltetrazolium bromide; BrdU: 5-bromo-2’-deoxy-uridine; AFP: Alpha-fetoprotein.

## Competing interests

The authors declare that they have no competing interests for writing this article.

## Authors’ contributions

BIC involved in interpretation of clinical data, conception and design of the translational research study in vitro and wrote the first draft of the manuscript. RD, MGR and CL participated equally at the design, execution and interpretation of the experiments. CM and AC provided overall supervision for conducting the study and involved in manuscript revision and presentation. AM performed in 3D Matrigel invasion experiments. All authors read and approved the final manuscript.

## Pre-publication history

The pre-publication history for this paper can be accessed here:

http://www.biomedcentral.com/1471-2407/14/43/prepub
